# Dynamic estimates of survival in oncocytic cell carcinoma of the thyroid

**DOI:** 10.1007/s12672-023-00839-4

**Published:** 2023-11-30

**Authors:** Yang Shi, Yuenan Zheng, Hao Zhang, Wenwu Dong, Ping Zhang

**Affiliations:** Department of Thyroid Surgery, The First Hospital of China Medical University, 155 Nanjing Bei Street, Shenyang, Liaoning People’s Republic of China

**Keywords:** Oncocytic cell carcinoma, Prognosis, Annual hazard, Conditional survival

## Abstract

**Background:**

Little is known about death hazard and conditional survival of oncocytic cell carcinoma of the thyroid (OCC).

**Methods:**

Patients diagnosed with OCC between 2004 to 2019 were obtained from the Surveillance, Epidemiology, and End Results (SEER) database. The Kaplan–Meier method was used to estimate the actuarial disease-specific survival (DSS). The annual hazard rate of death was depicted employing the hazard function. Based on the life-table method, the conditional DSS was calculated.

**Results:**

In terms of DSS rates, there were statistically significant differences among the different stages (*P* < 0.01). Annual hazard curves for mortality from OCC in the entire study participants demonstrated an overall decreasing tendency with two peaks at 3 and 10 years. In patients with distant disease, the death risk curve was the steepest and decreased quickly and evidently. Conditional DSS tended to increase over time in the entire study population. Patients with distant disease showed more significant alterations than those patients with local or regional disease.

**Conclusions:**

Prognosis improved over time in patients with OCC. The largest increase in conditional DSS was observed in patients with distant disease. Conditional survival may provide more relevant prognostic information than conventional survival estimates and allow personalized follow-up and counseling.

## Key points

Questions: How do the risk of death and survival probability change over time in patients with oncocytic cell carcinoma of the thyroid (OCC)?

Findings: In this retrospective study of 3401 patients, prognosis improves over time in patients with OCC of different Surveillance, Epidemiology, and End Results (SEER) stages. The greatest improvement was observed in patients with distant disease.

Meaning: For OCC survivors, death hazard and conditional survival analysis provide more accurate dynamic estimates of survival than conventional survival analysis for patients who have survived a period of time after diagnosis. With such information, clinicians can provide an accurate risk assessment of survival and formulate individual treatment and surveillance recommendations for patients diagnosed with OCC.

## Introduction

Oncocytic cell carcinoma of the thyroid (OCC) is uncommon, accounting for 3% of all thyroid carcinomas [1]. OCC superseded Hurthle cell carcinoma in the World Health Organization (WHO) classification for thyroid carcinoma in 2022 (5th edition) [1]. OCC is a separate organism that refers to oncocytic follicular cell-derived neoplasms (with more than 75% oncocytic cells) without the typical nuclear features of papillary thyroid carcinoma (PTC) and high-grade characteristics. Compared to non-oncocytic thyroid carcinomas, it has a higher level of aggressiveness [2]. The recurrence rate and mean duration to recurrence are correspondingly 12–33% and 90.74 months [3, 4]. In terms of disease-specific survival (DSS), the 5- and 10-year DSS are 85–95.4% and 92.6%, respectively [3, 5, 6]. However, whether multidisciplinary interventions in combination with radiation therapy and/or chemotherapy provide further survival benefits remains controversial [7, 8]. Due to the low morbidity and highly aggressive nature of OCC, patients are very concerned about their prognosis after diagnosis.

Currently, the prognostic evaluation of patients with OCC is limited to traditional actuarial DSS, there is a lack of conditional survival data, specifically for OCC, compared to the more common types of thyroid carcinoma. Further elaboration of the clinical relevance of dynamic prognostic tools for patients with OCC and healthcare providers would further enhance their rationale. Thus, we used death hazard and conditional DSS analyses to solve this problem [9, 10]. In the first approach, the death hazard at any given point among the remaining patients at risk is represented, which facilitates a clearer comprehension of time-specific mortality risks. The latter approach calculates the probability that a patient who has already survived for some time will survive for another period of time, providing a dynamic assessment of survival with increased survival time [9, 10]. This study aimed to evaluate the actuarial DSS, carcinoma mortality hazard rates, as well as conditional DSS in patients with OCC and further explore the effects of different Surveillance, Epidemiology, and End Results (SEER) stages.

## Materials and methods

This retrospective study used nationwide data from the SEER database of the National Cancer Institute, which collects and provides incidence and survival statistics from 18 population-based cancer registries encompassing approximately one-third of the US population. Thus, it could provide the large sample size spanning 15 years as a major strength, granting power to detect survival differences between subgroups. We used the histopathology codes of the International Classification of Disease for Oncology (3rd edition; ICD-O-3; code 8290/3: Oxyphilic adenocarcinoma) to identify patients diagnosed with OCC between 2004 and 2019. Patients with unknown SEER combined summary stage, no surgery, unknown survival times, or more than one type of primary carcinoma were excluded. Finally, 3401 patients with OCC were eligible for inclusion in this study. The following information was extracted from the SEER database: age at diagnosis, sex (male or female), race (white, black or other), SEER combined summary stage (localized, regional or distant), and treatment strategy (radiation therapy and chemotherapy). The SEER combined summary stage was used to define the disease stage as localized (restricted to the thyroid), regional (propagated beyond the thyroid to the surrounding tissue or lymph nodes), or distant (metastasized to other organs). The Institutional Review Board of the First Hospital of China Medical University deemed this study exempt from review because the data in the SEER database were anonymized and made freely accessible after obtaining permission.

## Analyses of statistical data

Continuous variables were presented as medians with interquartile ranges (IQRs). Categorical variables were described as counts and proportions. In this study, the main outcome was DSS, calculated from the time of diagnosis to the time of death due to OCC. Independent prognostic factors for DSS were determined using a Cox proportional hazard regression model. The Kaplan–Meier method was used to estimate DSS. Using the kernel smoothing method, hazard rates were inspected by means of a professional hazard assessment. The conditional DSS was defined as the probability that the patient would survive for another y years under the condition that he had already survived for x years. This can be described mathematically by the conditional DSS (x|y) = S (x + y)/S (x), where S (x) represents the overall survival rate over a period of x years. Conditional DSS was calculated according to the life table survival data. A two-sided test was performed on all data, with a *P* value less than 0.05. Statistical analyses were performed using SPSS software (version 26.0; IBM, Armonk, NY, USA) and the Stata statistical software package (version 16.0; Stata Corporation Ltd, College Station, TX, USA).

## Results

### Demographic and clinicopathological features

A summary of the baseline characteristics of the study population was shown in Table 1. The median age of the patients at diagnosis was 58 years. OCC was more common in females (68.5%) and in white patients (84.4%). According to the SEER combined stage system, most patients were classified with localized disease (84.3%), whereas only a minority of patients with regional (12.0%) and distant disease (3.7%). Radiation therapy and chemotherapy were administered to 1781 (52.4%) and 21 (0.6%) patients, respectively.

**Table 1:** Baseline characteristics of patients with OCC

Characteristics	No. of patients (%)
Age at diagnosis, median (IQR), years	58 (46–69)
Sex	
Female	2331 (68.5)
Male	1070 (31.5)
Race/ethnicity	
White	2872 (84.4)
Black	255 (7.5)
Others/unknown	274 (8.1)
SEER stage	
Localized	2866 (84.3)
Regional	409 (12.0)
Distant	126 (3.7)
Radiation therapy	
No	1620 (47.6)
Yes	1781 (52.4)
Chemotherapy	
No	3380 (99.4)
Yes	21 (0.6)

*OCC* oncocytic cell carcinoma, *IQR* inter quartile range, *SEER* combined summary stage

### Prognostic factors for DSS

Five factors were significantly associated with DSS in the univariate survival analysis, including age at diagnosis, sex, stage of the disease, treatment with radiation therapy, and chemotherapy (Table 2). Five factors were incorporated into a multivariate Cox proportional hazards regression model. Age at diagnosis, SEER stage, and chemotherapy were identified as the independent prognostic factors for DSS (Table 2).

**Table 2:** Univariable and multivariable Cox proportional hazards regression analyses of clinicopathological parameters associated with disease specific survival in patients with OCC

Variable	Univariate analysis		Multivariate analysis	
	HR (95% CI)	P	HR (95% CI)	P
Age at diagnosis	1.069 (1.055–1.083)	< 0.001	1.051 (1.038–1.065)	< 0.001
Sex				
Female	1.00 (reference)		1.00 (reference)	
Male	1.726 (1.249–2.384)	0.001	1.217 (0.876–1.690)	0.242
Race/ethnicity				
White	1.00 (reference)			
Black	0.501 (0.221–1.135)	0.097		
Others/unknown	0.877 (0.474–1.621)	0.674		
Seer stage				
Localized	1.00 (reference)		1.00 (reference)	
Regional	6.976 (4.630–10.510)	< 0.001	5.660 (3.730–8.587)	< 0.001
Distant	47.136 (32.154–69.098)	< 0.001	28.945 (19.364–43.265)	< 0.001
Radiation therapy				
No	1.00 (reference)		1.00 (reference)	
Yes	0.666 (0.484–0.917)	0.013	0.888 (0.642–1.229)	0.473
Chemotherapy				
No	1.00 (reference)		1.00 (reference)	
Yes	19.853 (10.074–39.125)	< 0.001	3.462 (1.693–7.077)	< 0.001

*OCC* oncocytic cell carcinoma, *HR* hazard ratio, *CI* confidence interval, statistically significant at P < 0.05

### Traditional actuarial DSS

**Fig. 1:**
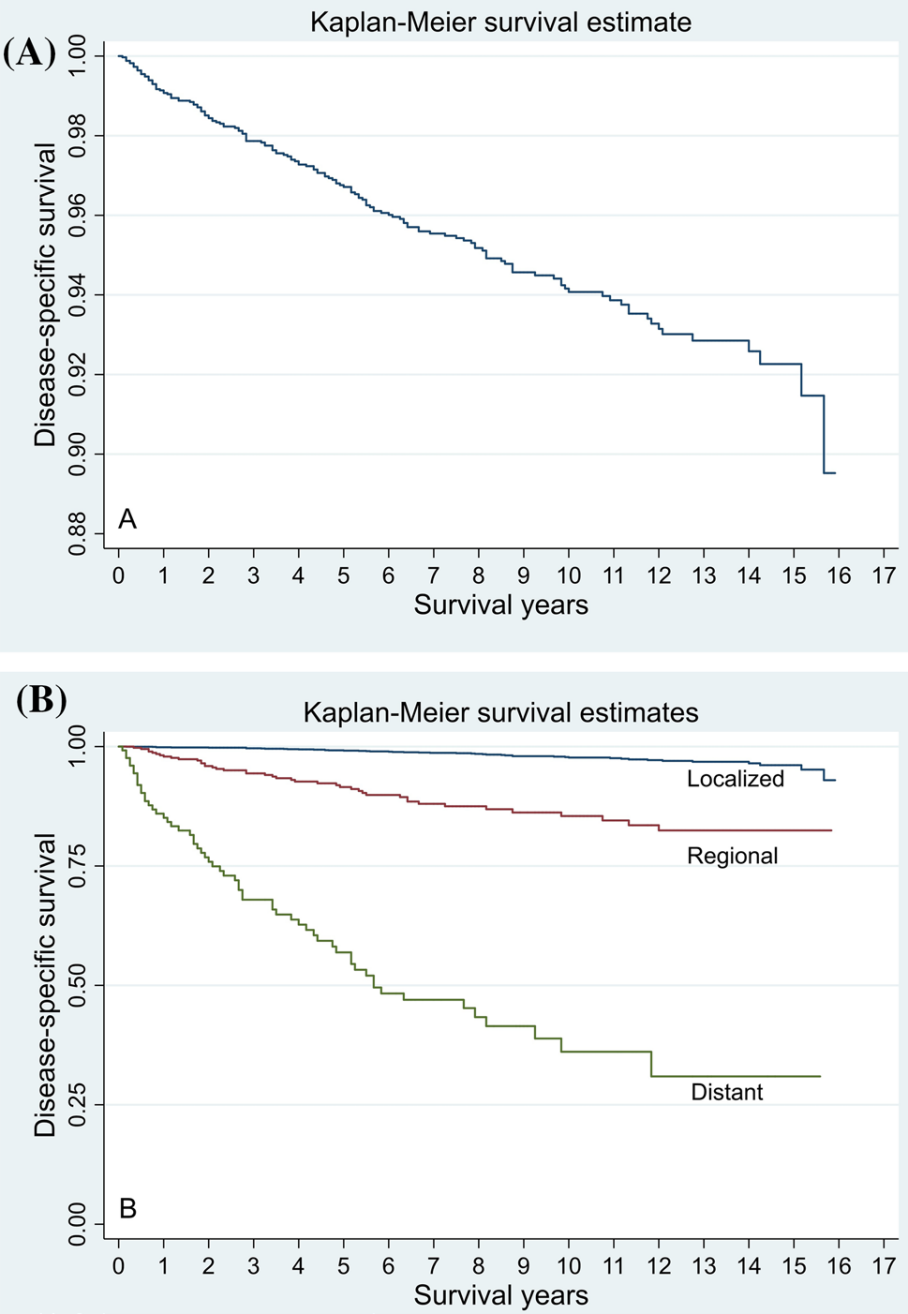
Kaplan–Meier curves for actuarial disease-specific survival of the entire cohort (**A**) and patients with different SEER stages (**B**)

The median follow-up duration was 86 months (40–133 months). Of the 3401 patients, only 152 (4.5%) died due to OCC during the follow-up period. The 1-, 5-, and 10-year DSS rates of the overall population were 99.1 ± 0.2%, 96.8 ± 0.3% and 94.2 ± 0.5%, respectively (Fig. 1). The 1-, 5-, and 10-year DSS rates among patients with distant, regional, and localized disease were 85.1 ± 3.2%, 97.9 ± 0.7%, 99.9 ± 0.1%; 56.9 ± 4.9%, 91.5 ± 1.6%, 99.2 ± 0.2%; 36.1 ± 5.9%, 85.5 ± 2.3%, 97.7 ± 0.4%, respectively. In terms of DSS rate, statistical significance was observed among the different stages of SEER (*P* < 0.01; Fig. 1).

### Death hazard analysis

**Fig. 2:**
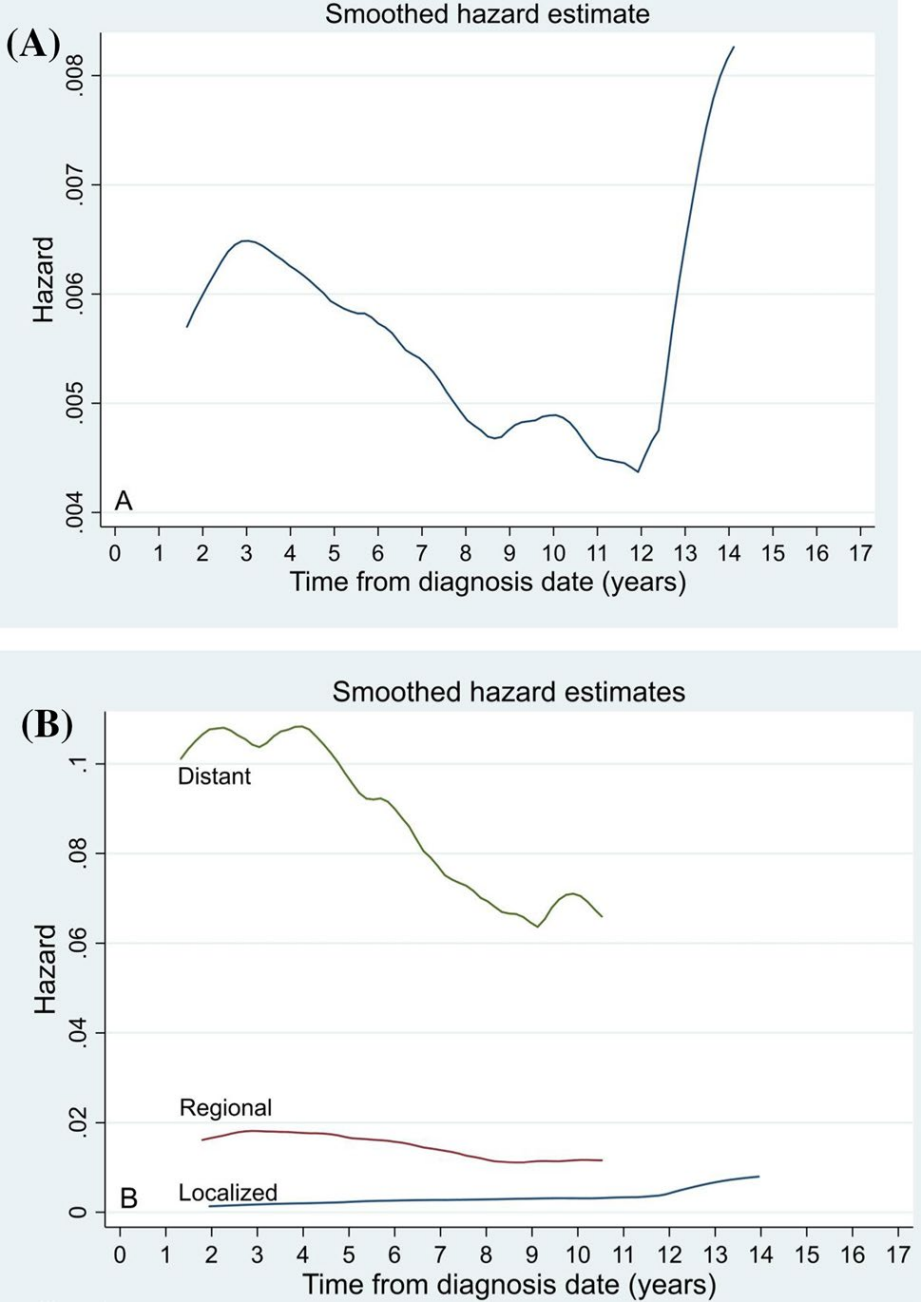
Annual hazard rate of carcinoma death for the entire cohort (**A**) and patients with different SEER stages (**B**)

The annual hazard curves for mortality from OCC in the entire study population showed an overall decreasing trend until 12 years after diagnosis, with two peaks at 3 and 10 years. In stratified analyses based on SEER stage, the death risk curve for patients with distant disease was the highest and declined more rapidly and clearly than those for patients with localized or regional disease. In general, the death risk curves for patients with localized and regional disease plateaued (Fig. 2).

### Conditional survival analysis

**Fig. 3:**
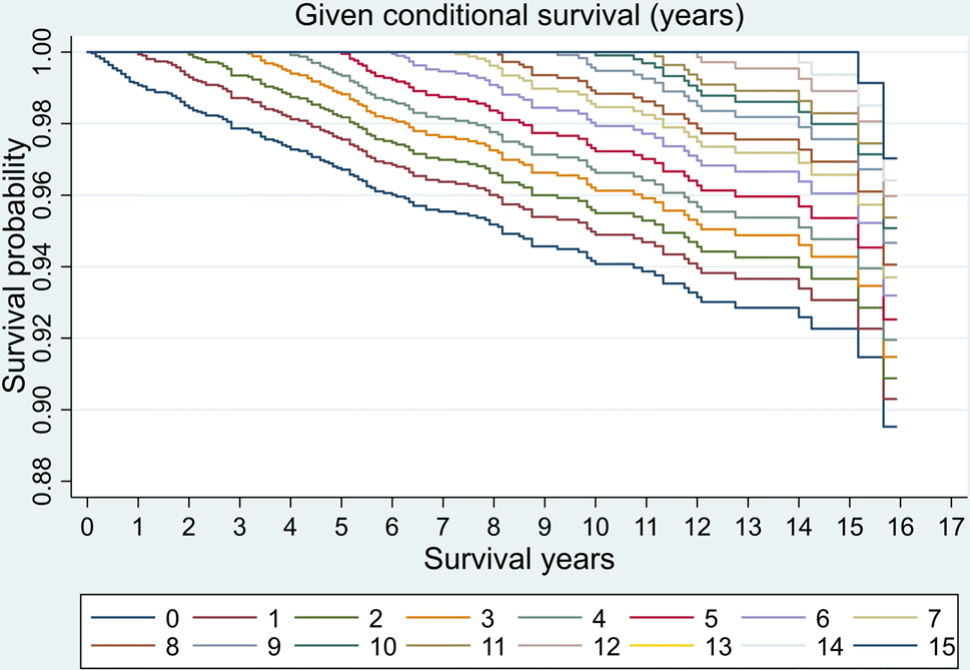
The probability of conditional DSS for patients who survive up to a certain time point given that they have already survived for several years

**Fig. 4:**
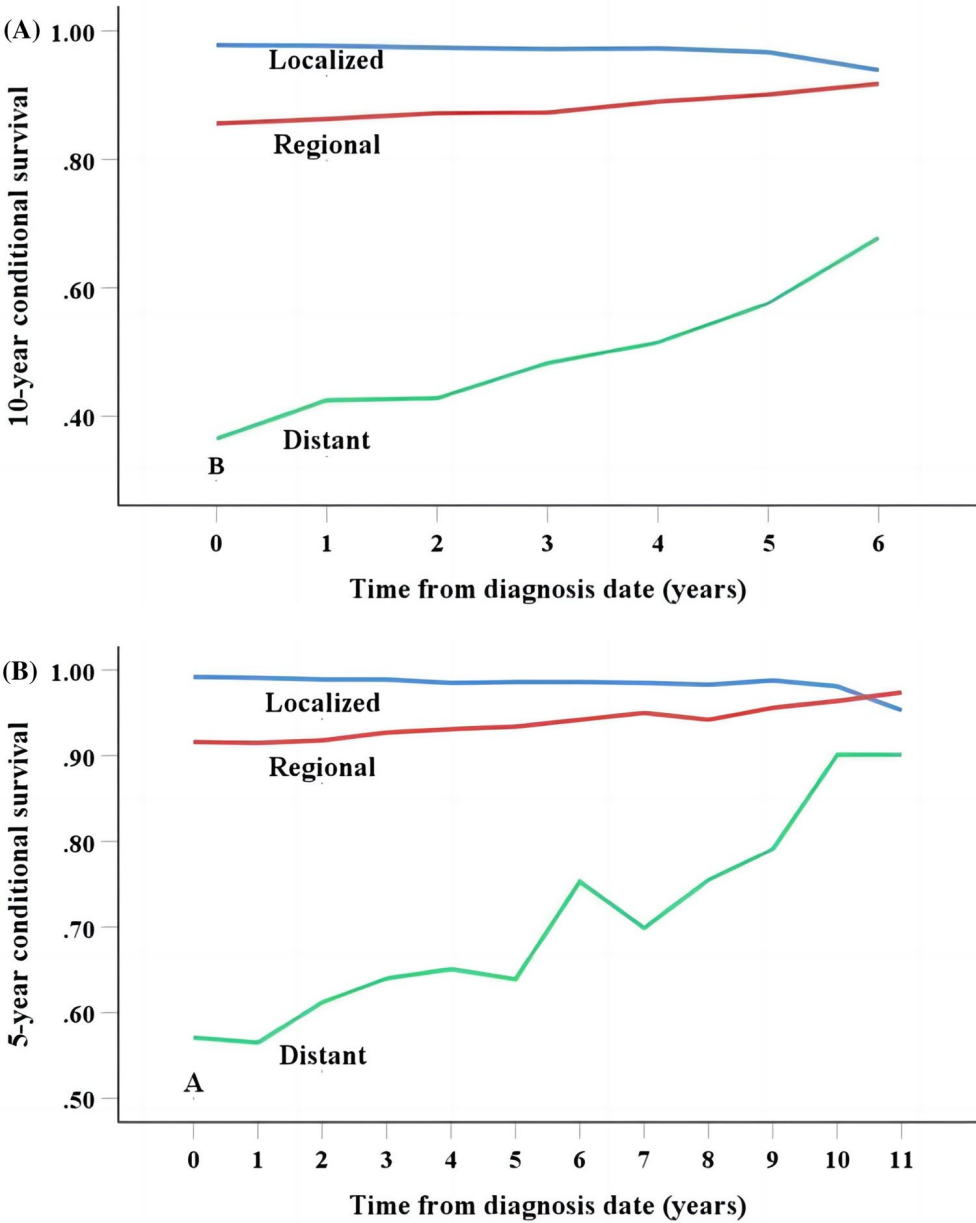
The 5- (**A**) and 10-year (**B**) conditional disease-specific survival at different time points for patients with different SEER stages

The conditional DSS of OCC patients was shown in Table 3 and Fig. 3. The conditional DSS increased each year relative to the overall survival time. Considering the possibility of the 1-year conditional DSS exceeded 99.1%, we calculated the 5- and 10-year conditional DSS, respectively. There was a rapid upward trend in the conditional DSS curves for the 5- and 10-year conditional DSS. In addition, the conditional DSS changed more significantly over time in patients with distant disease. The 5- and 10-year conditional DSS at different time points for patients with different SEER stages were shown in Fig. 4.

**Table 3:** The conditional disease-specific survival for OCC patients at a given time point

Total survival time (years)	Survival probability if patients have survived for (years, %)																
	0	1	2	3	4	5	6	7	8	9	10	11	12	13	14	15	16
0	100.0																
1	99.1	100.0															
2	98.5	99.4	100.0														
3	97.9	98.8	99.4	100.0													
4	97.4	98.3	98.9	99.5	100.0												
5	96.8	97.7	98.3	98.9	99.4	100.0											
6	96.1	97.0	97.6	98.2	98.7	99.3	100.0										
7	95.5	96.4	97.0	97.5	98.0	98.7	99.4	100.0									
8	95.2	96.1	96.6	97.2	97.7	98.3	99.1	99.7	100.0								
9	94.6	95.5	96.0	96.6	97.1	97.7	98.4	99.1	99.4	100.0							
10	94.2	95.1	95.6	96.2	96.7	97.3	98.0	98.6	98.9	99.6	100.0						
11	93.9	94.8	95.3	95.9	96.4	97.0	97.7	98.3	98.6	99.3	99.7	100.0					
12	93.3	94.1	94.7	95.3	95.8	96.4	97.1	97.7	98.0	98.6	99.0	99.4	100.0				
13	92.9	93.7	94.3	94.9	95.4	96.0	96.7	97.3	97.6	98.2	98.6	98.9	99.6	100.0			
14	92.9	93.7	94.3	94.9	95.4	96.0	96.7	97.3	97.6	98.2	98.6	98.9	99.6	100.0	100.0		
15	92.1	92.9	93.5	94.1	94.6	95.1	95.8	96.4	96.7	97.4	97.8	98.1	98.7	99.1	99.1	100.0	
16	89.6	90.4	91.0	91.5	92.0	92.6	93.2	93.8	94.1	94.7	95.1	95.4	96.0	96.4	96.4	97.3	100.0

## Discussion

In this study, we found that traditional actuarial DSS showed that patients with OCC had a high survival rate. Low DSS was associated with distant disease. The annual risk curve for mortality from OCC across the study population showed a downward trend, peaking at 3 and 10 years after diagnosis. The patients with distant disease showed a steeper curve than those with regional and localized disease. Conditional DSS showed an increasing trend with survival time. In short, we demonstrated that the improved survival probability depended heavily on the time elapsed after diagnosis. Patients with distant disease showed the most pronounced improvement.

Identifying prognostic factors facilitates the assessment of patient prognosis and the formulation of follow-up plans. Younger age at diagnosis, smaller tumor size, tumors confined to the thyroid, lower tumor stage, and no distant metastases at diagnosis had been shown to be associated with improved survival rates [5, 7, 11–14]. In a study by Zhou et al. [5] based on the SEER database, it was shown that distant SEER stage was an independent risk factor for death in OCC. In our study, we also found that SEER stage was the most influential prognostic factor among all independent prognostic factors. Therefore, we focused on the impact of different SEER stages on patient prognosis.

Traditional actuarial DSS for patients with OCC can provide information on the cumulative time distribution of carcinoma deaths. In a study of 239 patients [3], the 1-, 5-, 10-, and 20-year DSS rates for the overall population reached 98.0%, 94.6%, 92.5%, and 87.4%, respectively. In other studies [7], 10-year DSS rates ranged from 49.0% to 93.1%. Similarly, in our study, the prognosis of OCC patients was good. However, the DSS of patients with distant disease decreased significantly.

In our previous study, the hazard curve for PTC [15] showed a bimodal distribution with a small peak at 10 years, a trough at 14 years, and a major peak at 20 years after diagnosis in the whole population. In this study, the mortality from OCC hazard curve for the entire study cohort showed a bimodal pattern, with two peaks at 3 and 10 years. The hazard curve for (anaplastic thyroid carcinoma, ATC) in the entire study showed a decreasing trend, with an inflection point at 2.5 years after diagnosis and no significant peak [16]. This discrepancy may be due to the different types of malignancies.

In other studies of nasopharyngeal [17], esophageal [18, 19] and bladder carcinoma [20], the conditional DSS rates increased as the time to diagnosis progressed. Similar results were also observed in our study. For carcinoma studies with relatively good prognosis, such as gastric carcinoma [21], upper tract urothelial carcinoma [22], and bladder carcinoma [20], clinicians and patients were more concerned with 5-year, or even 10-year survival rates. In our research, patients with OCC had a probability of 1-year conditional DSS of more than 99.1% at 1, 2, 3, 5, or even 10 years after diagnosis. Therefore, we estimated the patients' conditional DSS at 5 and 10 years, respectively. The 5- and 10-year conditional DSS increased in OCC patients, with the greatest improvement observed in patients with distant disease. More importantly, the 10-year traditional actuarial DSS for patients with distant disease was only 36.5% compared to 63.9% at 5-year conditional 5 years after diagnosis. This suggests that if patients remain alive 5 years postoperatively, the probability of survival for another 5 years would increase from 36.5 to 63.9%. This dynamic observation of the patient's prognosis will provide a better understanding of the patient's prognosis and formulate more precise treatments to improve the patient's prognosis. This correlation also exists in other malignant tumors. Wang et al. [17] analyzed the survival of 1993 patients with non-metastatic nasopharyngeal carcinoma after intensity-modulated radiation therapy and found that the 5-year conditional DSS improved significantly in surviving patients with advanced-stage disease. Ploussard et al. [20] investigated the changes in the 5-year conditional DSS rates of 8141 patients treated with radical cystectomy for bladder carcinoma and found that improvements of 5- and 10-year conditional DSS were mainly noted in patients with advanced disease. In a study based on 7531 patients with melanoma [23], the same variation in conditional DSS rates was found in patients with different stages. For stage I patients, the 5-year conditional DSS increased from 61.7 to 93.2% if the patient survived for 4 years without recurrence and the stage II–IV patients had the similar trend. This improvement was more pronounced in stage IV patients [23]. Panunzio et al [24] also using the SEER database, the survival probability of patients with adrenocortical carcinoma treated with surgery was found to increase with the duration of the disease-free interval. Patients with stage III disease showed the most significant improvement [24]. The 3-year survival rate for colorectal liver metastasis patients with perineural invasion (PNI) [25] on pathological was 46.4% and the patients without PNI were 68.6%. The 3-year survival rate of patients with PNI was comparable to that of patients without PNI among those who lived 3 years postoperatively. These results suggest that for advanced malignant tumors, the conditional DSS improved significantly with increasing time since diagnosis.

### Limitation

First, the SEER database was limited to the U.S. population and may not be applicable to other populations. Second, due to the limitations of using registry data such as potential issues with coding inaccuracies or missing data, we were not precisely able to capture variables. Third, there is a lack of the duration, dose, or frequency of postoperative adjuvant therapy. Therefore, we were unable to interpret the impact of chemotherapy and radiation therapy on survival. Fourth, recurrence-free survival rates could not be analyzed because of the lack of relevant data. Thus, recurrence-free survival in OCC patients deserves further study. Fifth, there were only 126 cases of OCC patients with distant disease, and information about the number and location of distant metastases was limited; therefore, we were not able to further analyze the impact of the number and location of distant metastases on conditional DSS.

## Conclusion

Conditional survival of OCC patients was increased over time, especially for patients with distant disease. Thus, it is necessary to assess the long-term prognosis of OCC patients by the conditional survival analysis, which could provide a more valuable information and help clinicians to design individualized treatments for patients.

## Data Availability

The datasets generated during and/or analysed during the current study are available in the Surveillance, Epidemiology, and End Results (SEER) database, SEER Data & Software (cancer.gov).
